# Elevated autocrine chemokine ligand 18 expression promotes oral cancer cell growth and invasion via Akt activation

**DOI:** 10.18632/oncotarget.7585

**Published:** 2016-02-22

**Authors:** Xiao Jiang, Juan Wang, Xijuan Chen, Yun Hong, Tong Wu, Xiaobing Chen, Juan Xia, Bin Cheng

**Affiliations:** ^1^ Guangdong Provincial Key Laboratory of Stomatology, Guanghua School of Stomatology, Sun Yat-sen University, Guangzhou, Guangdong 510055, China; ^2^ Guangdong Provincial Stomatological Hospital, Guangzhou, Guangdong 510280, China

**Keywords:** oral squamous cell carcinoma, chemokine (C-C motif) ligand 18 (CCL18), proliferation, invasion, Akt

## Abstract

Chemokine (C-C motif) ligand 18 (CCL18) has been implicated in the pathogenesis and progression of various cancers; however, in oral squamous cell carcinoma (OSCC), the role of CCL18 is unknown. In this study, we found that CCL18 was overexpressed in primary OSCC tissues and was associated with an advanced clinical stage. CCL18 was found in both the cytoplasm and cell membrane of OSCC cells and was predominantly produced by cancer epithelial cells, as opposed to tumor-infiltrating macrophages. *In vitro* studies indicated that the effects of endogenous CCL18 on OSCC cell growth, migration, and invasion could be blocked by treatment with a neutralizing anti-CCL18 antibody or *CCL18* knockdown, while exogenous recombinant CCL18 (rCCL18) rescued those effects. Akt was activated in rCCL18-treated OSCC cells, while LY294002, a pan-PI3K inhibitor, abolished both endogenous and exogenous CCL18-induced OSCC cell invasion. *In vivo*, LY294002 treatment attenuated rCCL18-induced OSCC cell growth. Our results indicate that CCL18 acts in an autocrine manner via Akt activation to stimulate OSCC cell growth and invasion during OSCC progression. They also provide a potential therapeutic target for the treatment of oral cancer.

## INTRODUCTION

Oral squamous cell carcinoma (OSCC) accounts for approximately 90% of oral malignancies, with a 5-year survival rate of less than 50%, despite improved methods for diagnosis and therapy [1, 2]. OSCC arises from the surface epithelium of the oral cavity and progresses to carcinoma *in situ*, followed by invasive carcinoma, and finally metastatic carcinoma. Cancer cell growth and invasion are critical processes during OSCC development [3, 4]. Thus, it is essential to understand the mechanisms driving these processes.

Chemokines, a large family of cytokines that bind to specific G-protein-coupled receptors, participate in cancer development by activating downstream signaling pathways and affecting cellular behaviors [5–7]. Chemokine (C-C motif) ligand 18 (CCL18), a member of the CC chemokine subset, plays a crucial role in immune processes and inflammation by triggering biological responses in dendritic cells, fibroblasts, monocytes/macrophages, and cancer cells [8–10]. CCL18 is predominantly produced by M2 macrophages, and increased expression of CCL18 is observed in infiltrating macrophages in ovarian cancer, cutaneous T-cell lymphoma, gastric cancer, and breast cancer [11–14]. However, the origin of CCL18 may be cancer-type specific, because prostate cancer epithelial cells also secrete CCL18 [15]. Thus, CCL18 could act in an autocrine manner, paracrine manner, or both, during cancer development.

The role of CCL18 in cancer development also seems to vary among cancers. High CCL18 expression is correlated with prolonged patient survival in gastric cancer and colorectal cancer [13, 16]. In contrast, increased CCL18 expression indicates a poor prognosis in breast cancer [14]. The understanding of CCL18 function has been hampered until the recent identification of PITPNM3 (phosphatidylinositol transfer protein 3; also named PYK2 N-terminal domain interacting receptor 1, Nir1) in breast cancer, which has a high binding affinity for CCL18 [14]. We previously demonstrated that the mRNA expression of *CCL18* in oral premalignant lesions is 8.8-fold higher than that in normal oral mucosa [17]. However, *CCL18* expression in OSCC and its contribution to OSCC development have not been examined.

In the present study, we determined the expression and origin of CCL18 in OSCC tissue specimens and cell lines and analyzed its clinicopathological significance. Furthermore, we investigated the roles and downstream pathways of CCL18 in OSCC cell growth and invasion. Our findings demonstrate that elevated autocrine CCL18 accelerates cancer cell growth and invasion via Akt activation in OSCC.

## RESULTS

### CCL18 expression is upregulated in OSCC and positively correlates with advanced tumor stage

**Figure 1:**
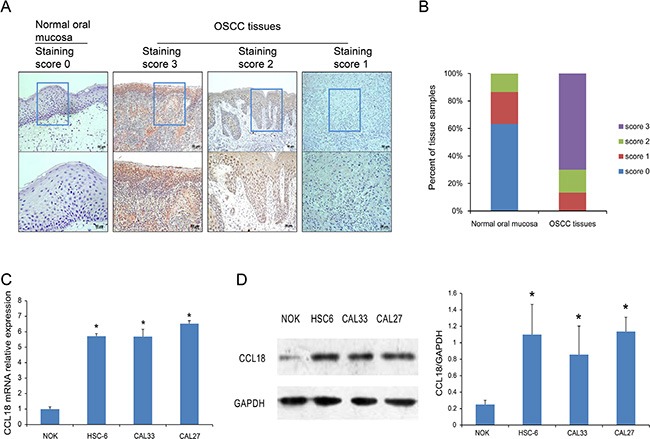
CCL18 protein and mRNA expression in OSCC tissues and cells (**A**) Representative images of CCL18 staining in normal oral mucosa with a staining score of 0 and OSCC tissues with staining scores of 3, 2 and 1. (upper panel, magnification 100 × ; lower panel, magnification 200 ×). (**B**) Quantitative evaluation of CCL18 expression in tissue samples of normal oral mucosa and OSCC based on the staining scores. (**C** and **D**) Quantitative PCR and western blotting assays for CCL18 expression in oral cancer cells (HSC-6, CAL33 and CAL27) and NOK cells. Columns represent the mean ± SEM of triplicate determinations. (*P < 0.05 vs. NOK cells).

To evaluate the expression of CCL18 in OSCC tissues, we used immunohistochemistry (IHC) to detect CCL18 protein in 60 OSCC tissues and 30 normal oral mucosa tissues. CCL18 expression was primarily located in the cytoplasm and cell membrane of oral cancer cells (Figure 1A). As shown in Figure 1B, compared with normal oral mucosa tissues, CCL18 expression was increased in OSCC tissues. All OSCC tissues displayed positive CCL18 expression, with 13.3% (8/60) displaying weak expression, 16.7% (10/60) displaying moderate expression, and 70.0% (42/60) displaying strong expression. We also identified a positive association between CCL18 expression and tumor TNM stage in OSCC patients (*P =* 0.040, Table 1). However, there were no correlations between CCL18 expression, patient age, gender, tumor site, histological differentiation, or lymph node metastasis.

**Table 1:** Clinicopathological association of CCL18 expression in oral squamous cell carcinoma

Characteristics	No.of cases	CCL18 staining score			*P* value
		1	2	3	
Age					0.767
< 50	26	4	3	19	
> 50	34	4	7	23	
Gender					0.923
Male	41	6	6	29	
Female	19	2	4	13	
Tumor site					0.152
Tongue	37	5	9	23	
Others	23	3	1	19	
T-primary tumor					0.159
T1 + T2	46	7	9	30	
T3 + T4	14	1	1	12	
N-regional lymph node					0.748
Non-Metastasis	48	6	8	34	
Metastasis	12	2	2	8	
Histological grade					0.907
Well	40	6	6	28	
Moderately + Poorly	20	2	4	14	
TNM stage					0.040
I + II	43	8	8	27	
III + IV	17	0	2	15	

To further confirm the increase in CCL18 expression in oral cancers, we examined the mRNA and protein levels of CCL18 in 3 OSCC cell lines (HSC-6, CAL33, and CAL27) and in normal oral keratinocytes (NOK). Compared with NOK cells, all OSCC cells had increased CCL18 mRNA (Figure 1C) and protein (Figure 1D) expression.

### Secretion of CCL18 from OSCC tissues and cell lines

**Figure 2:**
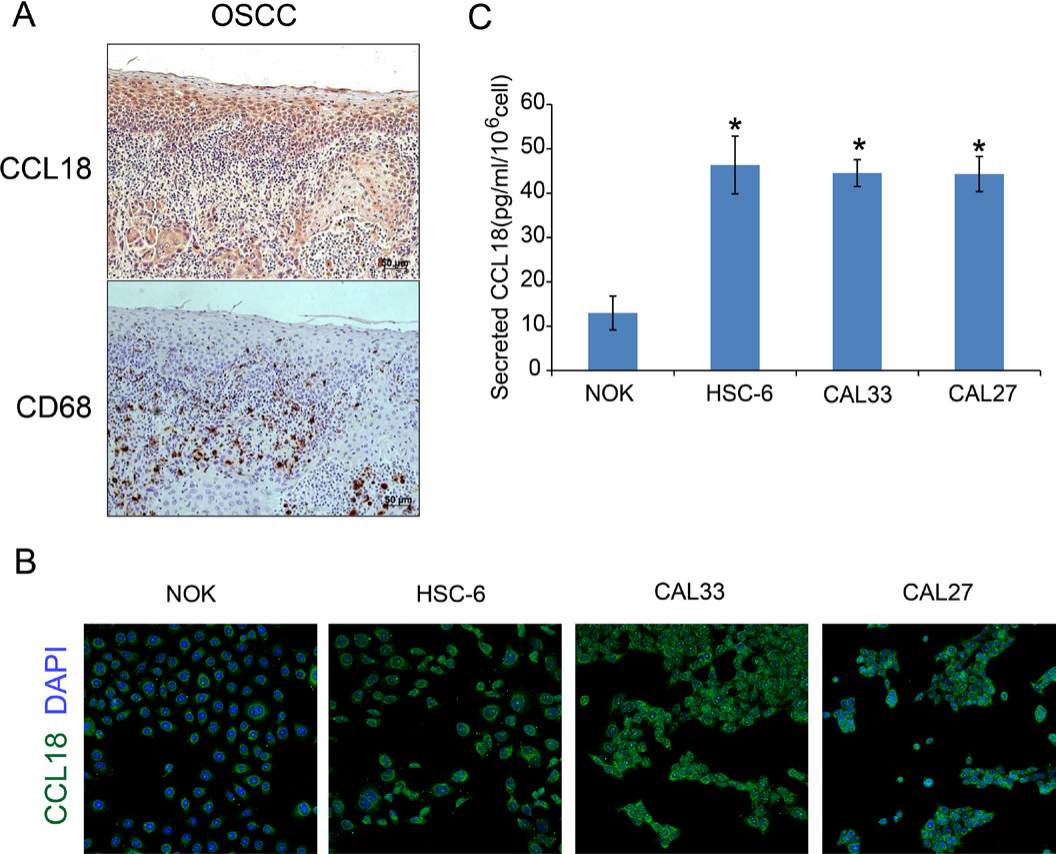
Secretion of CCL18 from OSCC tissues and cells (**A**) Representative consecutive OSCC tissue sections stained with anti-CCL18 and anti-CD68 antibodies and counterstained with hematoxylin (magnification 200 ×) (**B**) Immunofluorescence staining of CCL18 (green) in oral cancer cells (HSC-6, CAL33 and CAL27) and NOK cells. Nuclei were counterstained with DAPI (blue). (magnification 50 ×) (**C**) ELISA for CCL18 in the supernatant of oral cancer cells (HSC-6, CAL33 and CAL27) and NOK cells. The data represent the mean ± SEM of three independent experiments (*P < 0.05 vs. NOK cells).

We next asked which cells contribute to the increased chemokine CCL18 in OSCC. To examine CCL18 expression in tumor-associated macrophages (TAMs), consecutive OSCC tissue sections were used for IHC staining of the CCL18 protein and the macrophage marker CD68. CD68^+^ cells were located in the cancer stroma, while there was little co-localization of CD68^+^ and CCL18^+^ staining in OSCC tissues (Figure 2A). Immunofluorescence staining also indicated cytoplasmic and cell membrane staining of CCL18 in OSCC and NOK cells (Figure 2B). Furthermore, there was an increase in secreted CCL18 in OSCC cell supernatant as compared with NOK cell supernatant (Figure 2C). Taken together, these data provide evidence that elevated CCL18 in OSCC is attributed to cancer epithelial cells as opposed to TAMs.

### CCL18 promotes oral cancer cell growth *in vitro* and *in vivo*

**Figure 3:**
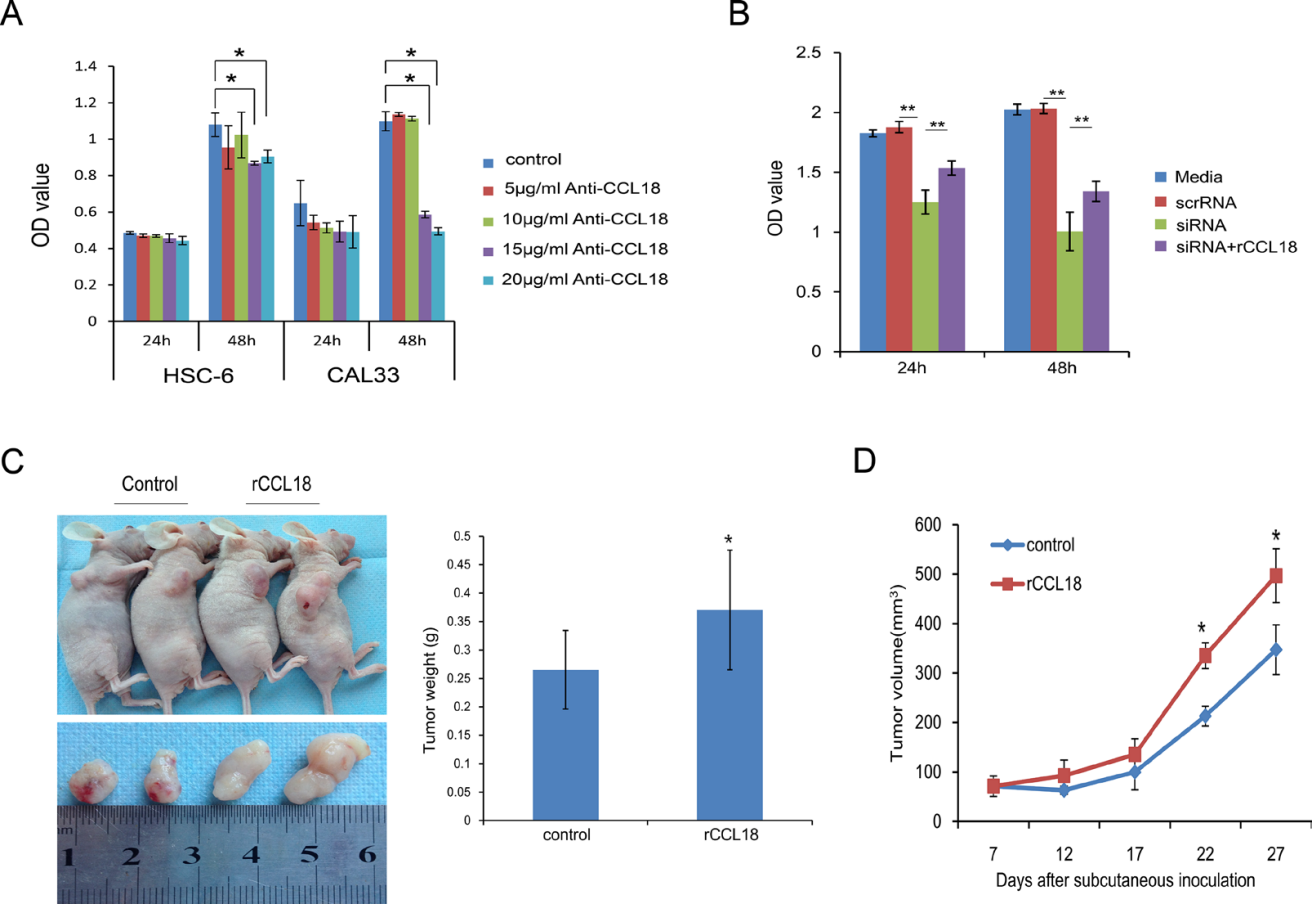
CCL18 promotes oral cancer cell growth *in vitro* and *in vivo* (**A**) OSCC cells (HSC-6 and CAL33) were treated with the indicated concentration of neutralizing CCL18 antibody (anti-CCL18). Cell viability was then measured using the CCK-8 assay at 24 h and 48 h. The data are presented as the mean ± SEM of triplicate experiments. (*P < 0.05) (**B**) HSC-6 cells were left untreated, transfected with 20 nM scrRNA, transfected with 20 nM siCCL18, or transfected with 20 nM siCCL18 and then treated with 20 ng/ml exogenous rCCL18 (siCCL18+rCCL18). Following treatment, cell viability was measured at 24 h and 48 h. The data are presented as the mean ± SEM of triplicate experiments (**P < 0.01). (**C** and **D**) Representative images of tumors obtained after subcutaneous injection of HSC6 cells into the flank region of nude mice, and treatment (3 times/week × 3 weeks) with vehicle control (*n =* 5) or exogenous rCCL18 (*n =* 6, 2 ng/g) starting on the 7th day. Tumor volumes were measured once every 5 days starting at the beginning of treatment. Four weeks after HSC-6 injection, the mice were sacrificed and the tumors were removed and weighed. Tumor weights (C) and volumes (D) are presented as the mean ± SEM. (*P < 0.05 vs. control).

To understand the biological significance of upregulated endogenous CCL18 in OSCC, we employed gain-of-function and loss-of-function studies in human OSCC cell lines. We used a CCL18 antibody (anti-CCL18 Ab) to neutralize secreted CCL18 in the supernatant and *CCL18* siRNA to knockdown endogenous *CCL18* in OSCC cells. Exogenous recombinant human CCL18 (rCCL18) was used to promote CCL18-induced effects. First, we used immunofluorescence, qRT-PCR, and western blotting to examine the expression of PITPNM3, the reported CCL18-specific transmembrane receptor, in OSCC cells. PITPNM3 was localized to the cell membrane and cytoplasm of OSCC and NOK cells (Supplementary Figure S1A). Neither mRNA nor protein expression of PITPNM3 differed between OSCC and NOK cells (Supplementary Figure S1B and S1C). We achieved efficient knockdown of CCL18 mRNA and protein using siCCL18–2 in HSC-6 cells (Supplementary Figure S2); as a result, siCCL18–2 was used in subsequent experiments. Depletion of secreted CCL18 in the supernatant with a neutralizing CCL18 antibody at a dosage higher than 15 μg/ml resulted in inhibition of HSC-6 and CAL33 cell growth after 48 h (Figure 3A). Similarly, transfection of *CCL18* siRNA led to a reduction in the growth rate of HSC-6 cells (Figure 3B). However, inhibition of cell growth by *CCL18* siRNA could be rescued by treatment with exogenous rCCL18 (Figure 3B). To further confirm the role of CCL18 in promoting oral cancer cell growth, a subcutaneous tumor formation assay was performed in BALB/C nude mice. As shown in Figure 3C and 3D, tumor growth was increased in the CCL18 group compared with the control group, as evidenced by the increased weight and volume of HSC-6 subcutaneous xenografts. Collectively, these observations indicate that CCL18 accelerates oral cancer cell growth *in vitro* and *in vivo*.

### CCL18 enhances oral cancer cell migration and invasion *in vitro*

**Figure 4:**
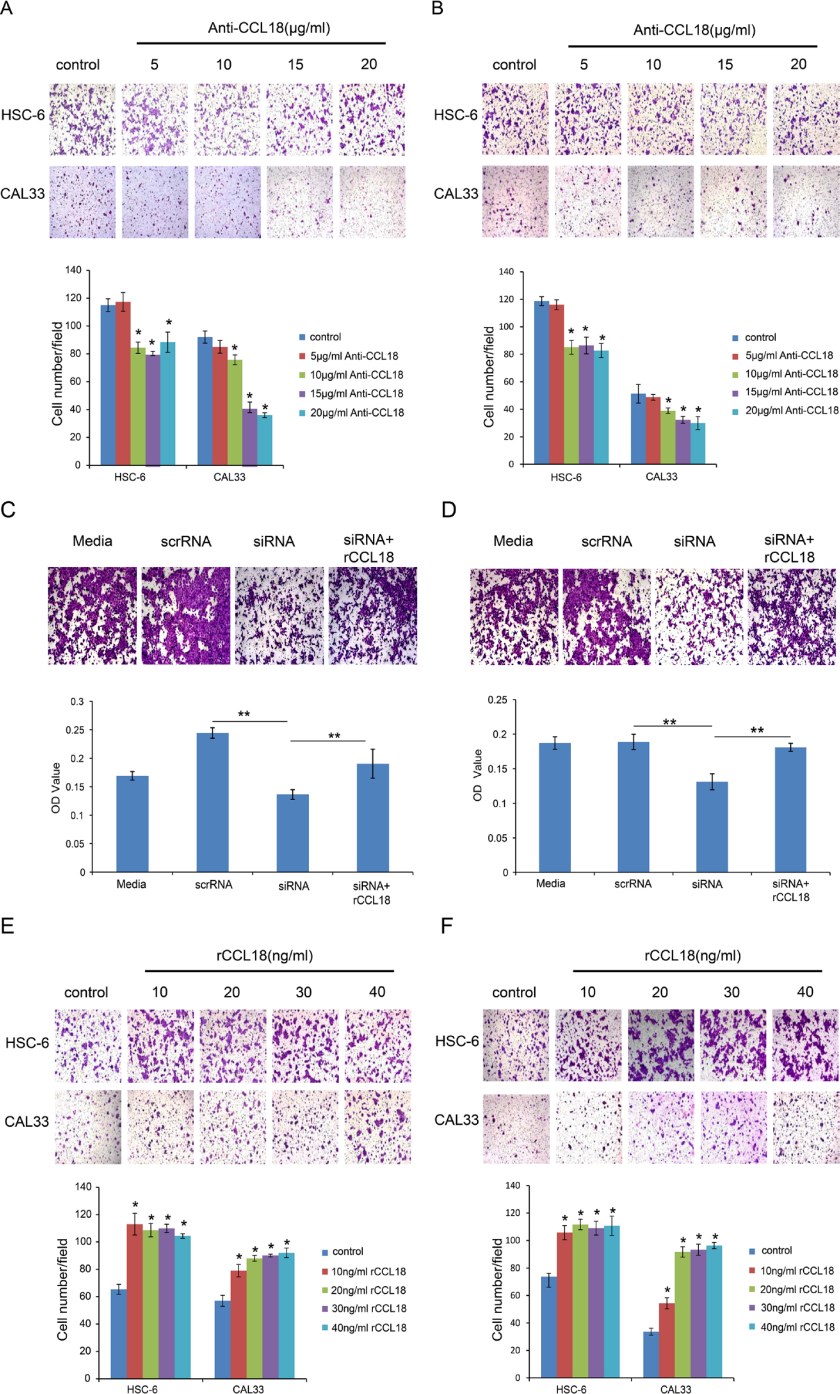
CCL18 enhances OSCC cell migration and invasion (**A** and **B**) The migration and invasion abilities of HSC-6 and CAL33 cells treated with the indicated concentrations of anti-CCL18 were evaluated by transwell migration and invasion assays. Representative pictures and the mean number of cells that migrated (A) or invaded (B) to the lower surface of three independent experiments (± SEM) are shown. (*P < 0.05 vs. control) (**C** and **D**) The migration and invasion abilities of HSC-6 cells were measured by transwell assay after the following treatments: transfection with 20 nM scrRNA, transfection with 20 nM siCCL18, or transfection with 20 nM siCCL18 and treatment with 20 ng/ml exogenous rCCL18 (siCCL18 + rCCL18). Representative pictures are shown and the mean OD values (± SEM) of migratory (C) or invasive (D) cells were measured at 590 nm. (*P < 0.05, **P < 0.01). (E and F) The migration and invasion abilities of HSC-6 and CAL33 cells treated with the indicated concentrations of exogenous rCCL18 were evaluated. Representative pictures and the mean number of migrated (**E**) or invaded (**F**) cells of three independent experiments (± SEM) are shown. (*P < 0.05 vs. control).

Clinicopathological analyses demonstrated that CCL18 expression is positively correlated with advanced tumor stage in OSCC patients (Table 1). Because invasion is essential for the development and metastasis of OSCC, we sought to characterize the effects of CCL18 on the migration and invasion of OSCC cells using transwell assays. To neutralize CCL18 function in oral cancer cells, we used both a CCL18 antibody as well as *CCL18* siRNA. In the presence of an anti-CCL18 antibody at concentrations above 10 μg/ml, both migratory (Figure 4A) and invasive (Figure 4B) HSC-6 and CAL33 cell numbers were reduced. Similar results were observed when HSC-6 cells were transfected with siCCL18 (Figure 4C and 4D). Exogenous rCCL18 rescued the inhibition in cell migration (Figure 4C) and invasion (Figure 4D) caused by siCCL18 transfection in HSC6 cells. In addition, treatment of HSC-6 and CAL33 cells with exogenous rCCL18 (10–40 ng/ml) enhanced cell migration and (Figure 4E and 4F). These data suggest that CCL18 stimulates oral cancer cell motility.

### CCL18 activates Akt signaling in OSCC cells

**Figure 5:**
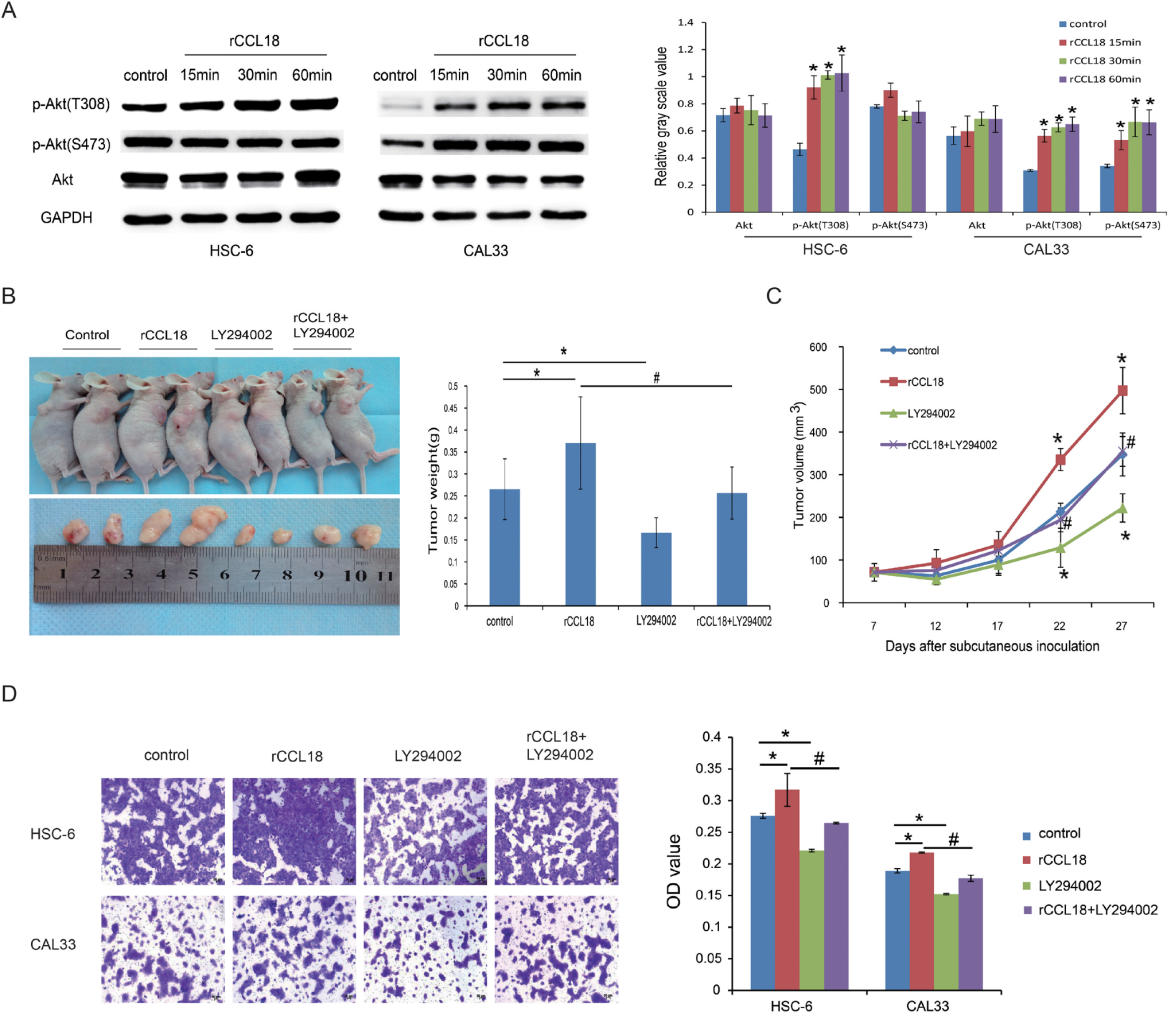
CCL18 activates Akt signaling in oral cancer cells (**A**) HSC6 and CAL33 cells were treated with 20 ng/ml exogenous rCCL18 for 15 min, 30 min, and 60 min. The cells were then harvested for western blotting to detect total and phosphorylated Akt (p-Akt) proteins. Densitometry was used to determine p-Akt/GAPDH and Akt/GAPDH ratios. Data represent mean ± SEM of three independent experiments. (*P < 0.05 vs. cells without treatment) (**B** and **C**) HSC-6 xenografts were treated (3 times/week × 3 weeks) with vehicle control (*n =* 5), rCCL18 (*n =* 6, 2 ng/g), LY294002 (*n =* 6, 100 ug/g) or their combination (*n =* 6, rCCL18+LY294002) starting on the same day of grouping. Tumor volumes were then measured once every 5 days. At the end of 3 weeks of treatment, the mice were sacrificed and the tumors were removed and weighed. Tumor weights (B) and volumes (C) are presented as the mean ± SEM. (*P < 0.05 vs. control, ^#^ *P* < 0.05 vs. rCCL18 group) (**D**) Invasion abilities of HSC-6 and CAL33 cells after treatment with vehicle control, 20 ng/ml rCCL18, 10 μmol/L LY294002, or their combination (rCCL18+LY294002) were evaluated. Representative pictures and the mean invaded cell numbers of three independent experiments (± SEM) are shown. (*P < 0.05 vs. control, ^#^*P* < 0.05 vs. rCCL18 group).

PI3K/Akt signaling has been associated with oral cancer progression [18, 19]. Therefore, we assessed whether Akt activation was involved in CCL18 autocrine signaling in oral cancer. We first examined the activation of Akt in rCCL18-treated HSC-6 and CAL33 cells. As shown in Figure 5A, treatment with 20 ng/ml of rCCL18 increased phosphorylated Akt (p-Akt) (Thr308) in HSC-6 cells and both p-Akt (Thr308) and p-Akt (Ser473) in CAL33 cells, while the total Akt protein in these two cell lines was unaffected. LY294002, a pan-PI3K inhibitor, was used to abolish CCL18-induced Akt activation and observe the effects on oral cancer cell growth and invasion. As shown in Figure 5B and 5C, although exogenous rCCL18 stimulated the growth of HSC-6 xenografts, this effect was attenuated by LY294002 treatment, as measured by both tumor size and weight. Similarly, LY294002 alone inhibited the growth of HSC-6 xenografts. Moreover, in a transwell assay LY294002 blocked both endogenous and exogenous CCL18-induced effects on HSC-6 and CAL33 cell invasion (Figure 5D).

## DISCUSSION

Chemokines in the tumor microenvironment play important roles in cancer development [20–22]. Aberrant expression of the chemokine CCL18 has been associated with several types of cancers, whereas its role in OSCC progression has not been studied. In this study, we demonstrate that elevated autocrine CCL18 facilitates cancer cell growth and invasion via Akt activation during the development of OSCC.

CCL18 is primarily expressed in monocytes, macrophages, and dendritic cells [8–10]. Excessive production of CCL18 in tumor-infiltrating macrophages was demonstrated in gastric cancer, breast cancer, colorectal cancer, and cutaneous T-cell lymphoma via CCL18 and CD68 co-immunostaining [13, 14, 16, 23]. Notably, IHC of human pancreatic ductal adenocarcinoma and prostate cancer revealed that both cancer epithelial cells and mesenchymal macrophages express CCL18 [15, 24]. Although elevated CCL18 in body fluids was reported in patients with lung cancer, bladder cancer, and ovarian cancer, the origin of CCL18 was not determined [25, 26, 27]. In the present study, we provide evidence that CCL18 is primarily overexpressed in the cytoplasm and cell membrane of OSCC cells and that little colocalization is observed between CD68^+^ and CCL18^+^ staining in OSCC tissues, indicating that the increased CCL18 in oral cancer is predominantly produced by cancer epithelial cells, as opposed to TAMs. Therefore, in oral cancer, CCL18 likely acts in an autocrine manner.

Both the origin and role of CCL18 varies with cancer type. Increased CCL18 is an independent favorable prognostic biomarker in patients with colorectal cancer and gastric cancer [13, 16]. In contrast, in patients with breast cancer, pancreatic cancer, cutaneous T-cell lymphoma, and lung cancer, elevated CCL18 in cancer tissues or body fluid indicates a worse clinical outcome [14, 23–25]. Consistent with this, we found that upregulated CCL18 in primary OSCC tissues is associated with advanced clinical stage.

Chemokines exert their effects by binding to specific transmembrane receptors. However, until recently, the CCL18 receptor had not been found. To date, three receptors have been proposed for CCL18: PITPNM3, GPR30 (G protein-coupled receptor 30), and CCR8 [14, 28, 29]. Only PITPNM3 has been related to cancer and only breast cancer cells have been reported to express PITPNM3. PITPNM3 overexpression was reported in breast cancer tissue and cancer cell lines, independent of the CCL18^+^ TAM counts [14]. Interestingly, almost no PITPNM3 expression was detected in gastric cancer tissue samples by IHC [14]. This disparity in PITPNM3 expression between breast and gastric cancers indicates that the two malignancies may respond differently to CCL18 and explains their distinct clinical outcomes. Our results demonstrate that in oral cancer cells, PITPNM3 is localized to the cell membrane and cytoplasm, and there is no difference in expression between cancer cells and normal oral epithelial cells, suggesting that CCL18 exerts its functions in oral cancer cells independent from PITPNM3. Furthermore, we found that both endogenous and exogenous CCL18 enhances human OSCC growth and invasion. CCL18 also accelerated cell invasion and metastasis in breast and lung cancer cells [14, 30–32]. The data presented here indicate that elevated CCL18 promotes OSCC development by endowing cancer cells with a more malignant phenotype.

PI3K/Akt signaling is correlated with OSCC development and progression [18, 33, 34]. We explored intracellular signaling involved in CCL18-induced OSCC malignant behaviors. We found that Akt is activated in CCL18-treated OSCC cells. LY294002, a pan-PI3K inhibitor, blocks both endogenous and exogenous CCL18-mediated effects on OSCC cell growth and invasion. However, the stimulatory effects of CCL18 on the growth and invasion of OSCC cells is not completely diminished by LY294002 treatment, implying that other signaling effectors may be triggered by the interaction between CCL18 and PITPNM3. Previous studies reported that CCL18-PITPNM3 binding induces Pyk2/Src or PI3K/Akt/GSK3β/Snail signaling and the subsequent metastasis of breast cancer [35]. All of these results indicate that CCL18 signaling might involve multiple intracellular pathways. Therapies targeting PI3K/Akt signaling have demonstrated impressive anticancer activities against a broad range of human cancers [36, 37]; therefore, agents blocking CCL18-induced PI3K/Akt signaling might provide complementary effects in OSCC treatment.

In summary, increased chemokine ligand CCL18, predominantly produced by cancer epithelial cells, is associated with advanced tumor stage in OSCC. Moreover, CCL18 contributes to cancer cell growth and invasion in an autocrine manner via Akt activation during OSCC progression. Our findings provide new insights into the role of CCL18 in cancer and new therapeutic targets for future OSCC treatment.

## MATERIALS AND METHODS

### Patients and samples

A total of 60 patients with oral squamous cell carcinoma (OSCC), who underwent surgical resection at the Department of Craniofacial Surgery, Guanghua School of Stomatology, SunYat-sen University, were enrolled in the study. Primary OSCC tissues (*n =* 60) and some adjacent normal tissues (*n =* 30) were obtained postoperatively. Informed consent was obtained from all patients. The study was approved by the Ethics Committee of Guanghua School of Somatology, Sun Yat-sen University.

### Cell lines and reagents

The OSCC cell line, CAL27, was purchased from American Type Culture Collection (Manassas, VA, USA). The OSCC cell lines (HSC-6 and CAL33) and normal oral keratinocytes (NOK) were kindly provided by J. Silvio Gutkind (NIH, Besthesda, MD). HSC-6, CAL33 and CAL27 cells were cultured in Dulbecco's modified Eagle's medium (DMEM, Gibco, Inc., Grand Island, NY, USA) supplemented with 10% fetal bovine serum (FBS). NOK cells were maintained in KSFM (Gibco, USA). Cells were incubated in a 5% CO2 atmosphere at 37°C. A neutralizing antibody against CCL18 (anti-CCL18) was purchased from R & D (R & D Systems, Minneapolis, MN, USA). Recombinant human CCL18 (rCCL18) was purchased from Peprotech (Peprotech, Inc., Princeton, NJ, USA).

### Immunohistochemistry (IHC)

Sections from paraffin-embedded OSCC or normal oral mucosa tissues were deparaffinized in xylene and rehydrated in a graded alcohol series. Antigen retrieval was performed by heat induction in citrate buffer, pH 6. The slides were incubated with primary antibodies against CCL18 (1:200, Abcam, Cambridge, MA, UK) or CD68 (1:100, ZSBio, Beijing, China) overnight at 4°C. After washing with PBST, the slides were incubated with HRP conjugated goat secondary antibody for 30 min. The color was developed using 3, 3′-diaminobenzidine (DAB). Finally, the slides were counterstained with hematoxylin.

The expression of CCL18 was quantified using a visual grading system based on the extent of staining (percentage of positive cells graded on a scale from 0 to 3: 0, < 5%; 1, 5–30%; 2, 30–70%; 3, > 70%) and the intensity of staining (graded on a scale from 0–3: 0, none; 1, weak staining; 2, moderate staining; 3, strong staining). Five representative fields at 400× magnification were evaluated. A weighted staining value (S) was obtained by multiplying the score for the percentage of positive cells by the score for the staining intensity. Then, a final score was assigned to each sample according to the S value: score 0 (negative), S = 0; score 1 (weak expression), S ≤ 2; score 2 (moderate expression), 3 ≤ S ≤ 4; score 3 (strong expression), 6 ≤ S ≤ 9.

### Reverse transcription PCR and quantitative real-time PCR

Total RNA was isolated with Trizol according to the manufacturer's protocol (Invitrogen, Inc., Carlsbad, CA, US). cDNA was synthesized using Transcriptor First Strand cDNA Synthesis Kit (Roche Applied Science). Quantitative real-time PCR was performed using the LightCycler^®^ 480 SYBR Green I Master and the LightCycler^®^ 480 instrument (Roche Applied Science) with the following primers: CCL18, 5′-TATGCCCAG CCACATTAACTAAC-3′ and 5′-GAAGGGAAAGGGGA AAGGA-3′; PITPNM3, 5′-TCGACATGGTGGCTCTG ACTG-3′ and 5′-ACATTGTATGTGATGCGACCACTG-3′; GAPDH, 5′-GCACCGTCAAGGCTGAGAAC-3′ and 5′-TGGTGAAGACGCCAGTGGA-3′. Expression of the transcripts were calculated relative to the level of the housekeeping gene GAPDH using the ^ΔΔ^Ct method. The assays were carried out in three independent trials.

### Western blotting

Cells were lysed with RIPA buffer (Sigma-Aldrich, Santa Clara, CA, USA). The protein levels of the lysates were measured with the BCA protein assay kit (Sigma-Aldrich, Santa Clara, CA, USA). Then, the proteins were separated by 10% SDS-PAGE and blotted to a PVDF membrane (Millipore, MA, USA). The membrane was blocked in 5% non-fat milk for 2 hours at RT and then incubated with primary antibody overnight at 4°C. Subsequently, the membrane was washed and incubated with HRP-conjugated secondary antibody for 1 hour. The immunoreactive bands were visualized with the enhanced chemiluminescence (ECL) detection system (Millipore, MA, USA). Immunoreactive bands were quantified by densitometry. Similar results were obtained in three independent experiments. The following primary antibodies were used: CCL18 (1: 2000, Abcam, Cambridge, MA, UK), PITPNM3 (1:1000, Santa Cruz, USA), and Akt (1:1000), p-Akt (T308, 1:1000), p-Akt (S473, 1:1000), and GAPDH (1:10000), which were all purchased from Cell Signaling (Beverly, MA, USA).

### Enzyme-linked immunosorbent assay

Cell supernatants were collected, and the levels of secreted CCL18 were measured using an ELISA kit (Abcam, Cambridge, MA, UK), according to the manufacturer's instructions.

### Immunofluorescence

Cells were plated on cover slips and subsequently cultured overnight. Then, cells were fixed with 4% formaldehyde, permeabilized with 0.1% Triton X-100 for 15 min, and blocked with 5% bovine serum albumin (BSA) for 1 h. Cells were incubated with primary antibodies CCL18 (1:100, Santa Cruz, USA) or PITPNM3 (1:100, GeneTex, USA) overnight at 4°C, followed by Dylight^®^ 488-conjugated or Dylight^®^ 594-conjugated secondary antibodies (1:100; Abcam, Cambridge, MA, UK) protected from light for 1 h at 37°C; subsequently, cells were counterstained with DAPI (ready-to-use, Invitrogen, Carlsbad, CA, USA) for 15 min. The cover slips were then observed under a confocal laser scanning microscope (Carl Zeiss AG, Germany).

### Small interfering RNA (siRNA)

*CCL18* siRNA (siCCL18) and scrambled siRNA (scrRNA) were designed by and purchased from Life Technologies. Two different *CCL18* siRNA duplexes were tested, and the sequences for siCCL18–1 and siCCL18–2 were as follows: 5′-ACAAGUUGGU ACCAACAAAdTdT-3′ and 5′-GAGCUGCAUUAUGAA AUUAdTdT-3′, respectively. Cells were seeded in 6-well plates or 96-well plates for 24 h and then transfected with siRNAs using the Lipofectamine^®^RNAiMAX reagent according to the manufacturer's instruction (Life Technologies, Carlsbad, CA, USA).

### CCK-8 cell viability assay

Cells were seeded into 96-well plates and then treated with the following: neutralizing CCL18 antibody (5, 10, 15 or 20 μg/ml), rCCL18 (10, 20, 30 or 40 ng/ml), 20 nmol/l scrRNA, or a combination of 20 nmol/l siCCL18 and 20 ng/ml rCCL18 (siCCL18+rCCL18). Then, cell viability was detected with a Cell Counting Kit-8 (CCK-8) according to the manufacturer's instructions (Sigma-Aldrich, Santa Clara, CA, USA). The absorbance at 450 nm was detected using a microplate reader (Thermo Electron, USA). All experiments were performed in triplicate.

### Cell migration and invasion assays

Cellular migration and invasion were detected with a transwell assay (Corning, Toledo, OH, USA). For the invasion assay, the upper chamber was pre-coated with 50 μl of 20% growth factor-reduced Matrigel (Gibco, Grand Island, NY, USA). For the migration assay, the inserts remained uncoated. In both assays, the cells were plated in serum-free medium, and medium supplemented with 10% FBS was used as a chemoattractant in the lower chamber. The cells were incubated for 24 h, and the cells that did not migrate or invade through the pores were removed with a cotton swab. The cells that had migrated or invaded into the lower surface of the membrane were stained with crystal violet and counted. Fields of view at 50× magnification were randomly imaged and quantified using a light microscope (Carl Zeiss AG, Germany).

### *In vivo* tumor growth in nude mice

Animal experiments were approved by the Committee on the Ethics of Animal Experiments of the Sun Yat-sen University, China (Permit Number: 00054610). Four week-old female BALB/c nude mice were purchased from the Animal Care Unit of Guangdong (Guangdong, China). HSC-6 cells (5 × 10^6^ cells) were injected subcutaneously into the flank region of nude mice. After 7 days, all tumors reached a size of approximately 80 mm^3^, at which time the mice were randomized into different groups (*n =* 5–6/group) for the following treatments: vehicle control, rCCL18 (2 ng/g), PI3K inhibitor LY294002 (100 ug/g, Selleck, Houston, TX, USA), and their combination. All mice were treated 3 times/week × 3 weeks. Tumor volume (mm^3^) was measured using caliper measurements every five days and calculated using the formula, V = d^2^ × D/2, where d and D represented the shortest and longest diameters, respectively. Mice were killed 4 weeks after injection. The tumors were then removed and weighed for further analysis.

### Statistical analysis

Statistical analyses were performed using the SPSS16.0 software (SPSS, Chicago, Illinois, USA). Data are presented as the means ± SEM. Data were analyzed using Student's *t-*test, one-way analysis of variance, or the Chi-square test. *P* < 0.05 was considered statistically significant.

## SUPPLEMENTARY MATERIALS FIGURES


