# Supplementary material for: Elevated autocrine chemokine ligand 18 expression promotes oral cancer cell growth and invasion via Akt activation
Source: Oncotarget. 2016 Feb 22;7(13):16262–72. doi: 10.18632/oncotarget.7585 (PMC4941312; doi:10.18632/oncotarget.7585)
Supplement: Supplementary file 1 [file oncotarget-07-16262-s001.pdf]

Elevated autocrine chemokine ligand 18 expression promotes oral cancer cell growth and invasion via Akt activation

Supplementary Materials

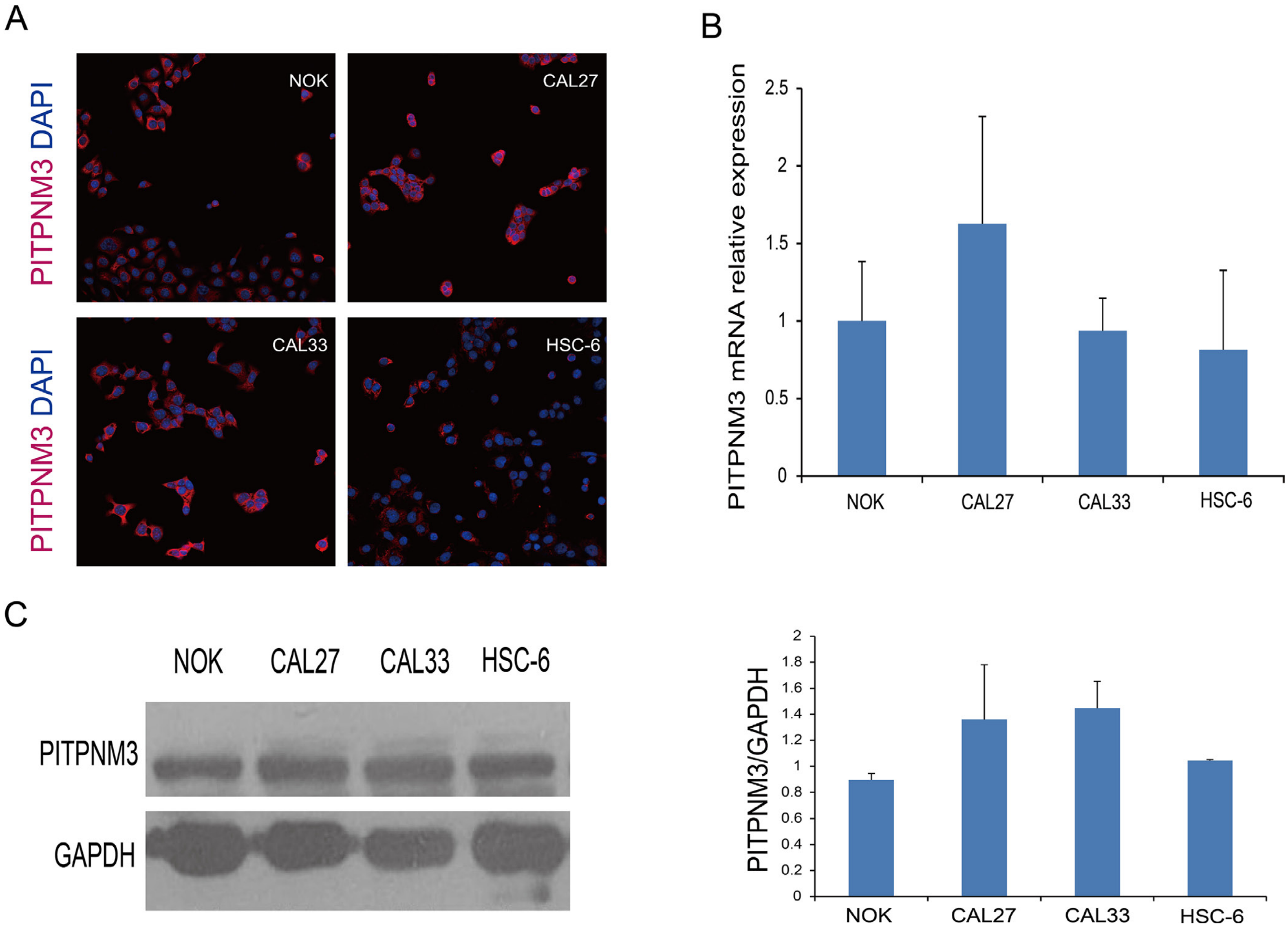

Supplementary Figure S1: Expression of PITPNM3 in OSCC and NOK cells. (**A**) Immunofluorescence staining of PITPNM3 (red) in oral cancer cells (HSC-6, CAL33 and CAL27) and NOK cells. Nuclei were counterstained with DAPI (blue). (magnification 50 ×) (**B** and **C**) Quantitative PCR and western blotting assays for PITPNM3 in oral cancer cells (HSC-6, CAL33 and CAL27) and NOK cells. Columns represent mean ± SEM of triplicate determinations.

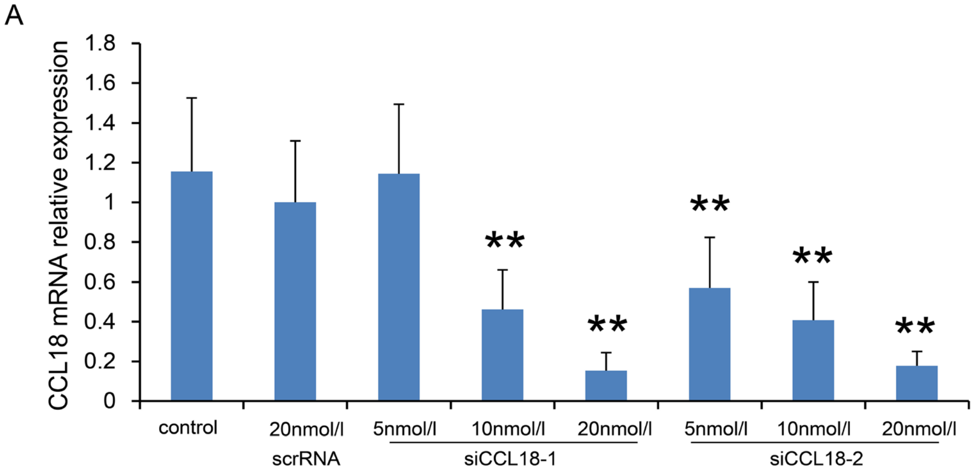

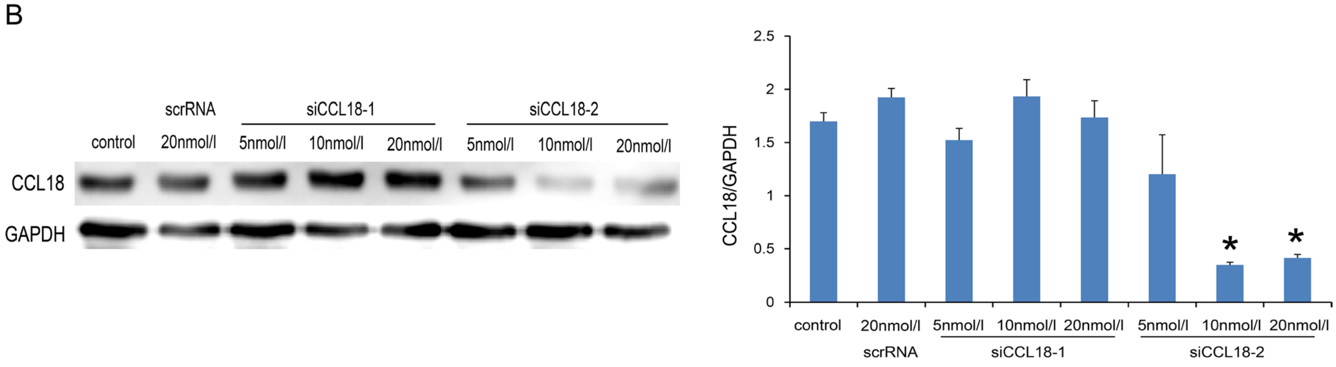

Supplementary Figure S2: Efficiency of CCL18 RNA interference in HSC-6 cells. HSC-6 cells were transfected with siCCL18–1 and siCCL18–2 respectively. After 48 h the cells were harvested. (**A**) Quantitative RT-PCR analysis of CCL18 mRNA levels. (**B**) Western blotting analysis of CCL18 protein levels. Densitometry was used to determine CCL18/GAPDH ratios. Data represent mean ± SEM of three independent experiments. (**P* < 0.05 and ***P* < 0.01 vs. cells transfected with scrRNA).
